# Integration measurement and its applications in low- and middle-income country health systems: a scoping review

**DOI:** 10.1186/s12889-023-16724-2

**Published:** 2023-09-28

**Authors:** Rachel Neill, Nukhba Zia, Lamisa Ashraf, Zainab Khan, Wesley Pryor, Abdulgafoor M. Bachani

**Affiliations:** 1 Department of International Health, Johns Hopkins Bloomberg School of Public Health, Johns Hopkins International Injury Research Unit, Health Systems Program, 615 N. Wolfe Street Suite E8527, Baltimore, MD 21205 USA; 2 Nossal Institute for Global Health, Melbourne School of Population and Global Health, The University of Melbourne, Melbourne, 3010 Australia

**Keywords:** Integrated care, Health systems, Low- and middle-income country, Measurement, Scoping review

## Abstract

**Background:**

Despite growing interest in and commitment to integration, or integrated care, the concept is ill-defined and the resulting evidence base fragmented, particularly in low- and middle-income countries (LMICs). Underlying this challenge is a lack of coherent approaches to measure the extent of integration and how this influences desired outcomes. The aim of this scoping review is to identify measurement approaches for integration in LMICs and map them for future use.

**Methods:**

Arksey and O’Malley’s framework for scoping reviews was followed. We conducted a systematic search of peer-reviewed literature measuring integration in LMICs across three databases and screened identified papers by predetermined inclusion and exclusion criteria. A modified version of the Rainbow Model for Integrated Care guided charting and analysis of the data.

**Results:**

We included 99 studies. Studies were concentrated in the Africa region and most frequently focused on the integration of HIV care with other services. A range of definitions and methods were identified, with no single approach for the measurement of integration dominating the literature. Measurement of clinical integration was the most common, with indicators focused on measuring receipt of two or more services provided at a single point of time. Organizational and professional integration indicators were focused on inter- and intra-organizational communication, collaboration, coordination, and continuity of care, while functional integration measured common information systems or patient records. Gaps were identified in measuring systems and normative integration. Few tools were validated or publicly available for future use.

**Conclusion:**

We identified a wide range of recent approaches used to measure integration in LMICs. Our findings underscore continued challenges with lack of conceptual cohesion and fragmentation which limits how integration is understood in practice.

**Supplementary Information:**

The online version contains supplementary material available at 10.1186/s12889-023-16724-2.

## Background

Integration has gained prominence as an approach to strengthen health systems towards Universal Health Coverage (UHC) [1–4]. Specific to low- and middle-income countries (LMICs), integration is frequently focused on the integration of vertical programs for specific disease or service packages for specified populations, including HIV/AIDS, tuberculosis (TB), and reproductive health care [4, 5], with the goal of increasing access to and coverage of prioritized services [6, 7]. Recommendations for integration in LMIC health systems are often based on normative policy guidance, with little evidence on the effectiveness of integration to improve health outcomes.

Underlying this challenge is a scarcity of evidence on measuring and evaluating integration as an intermediate outcome. This results in a weak understanding of the contextual complexities associated with integration reforms, constrains implementation of integrated approaches, and limits the generalizability of outcomes to improve health services [5, 6, 8–11].

### Existing measurement approaches for integration

We identified three previous reviews on the measurement of integration. A 2009 systematic review of integrated health care delivery identified 24 different methods, mainly focused on structural and process aspects [12]. A 2016 systematic review identified 209 index instruments, 84% of which were focused on clinical integration [13]. Finally, a 2017 knowledge synthesis review for health systems integration located 114 unique tools [14], primarily measuring care coordination, patient engagement, and team engagement [14].

Considered together, these findings indicate that while the available measurement approaches in published literature have increased, there remains a lack of cohesiveness and conceptual maturity in constructs, definitions, and measurement approaches. People-centered care and clinical integration dominate the literature, with most evidence originating from high-income countries (HICs). Few tools have been validated in LMICs [12–14].

### Rationale

A scoping review methodology is appropriate when the goal is to map an evolving field for the purpose of dissemination to practitioners and to identify gaps [15]. A search for “integrated health service delivery” on PubMed shows rapid growth of published studies since 2014. A scoping review of integration measurement approaches is warranted to capture new measurement tools, document where conceptual maturity has evolved, and identify gaps.

We are specifically motivated by a practical example – how to measure the integration of rehabilitation into health systems? Recent recommendations to integrate rehabilitation services into health systems have been made on conceptual merits, without a requisite understanding of what integration means or how to measure when it has been achieved [16]. Conceptually, rehabilitation is well positioned for an integrated approach, requiring services across the life course [17]. There is limited access to rehabilitative care in many LMIC health systems and an opportunity to improve access to services through an integrated, rather than verticalized, approach. Understanding how other scholars have measured integration can both support the development and measurement of integration of rehabilitative care into health systems and generate lessons for other health services.

We have focused our review on LMIC settings for two reasons. First, there are continued gaps in the evidence on integration approaches in LMICs [4, 18]. Second, the differing integration models in LMICs versus HICs warrants specific exploration of measurement approaches relevant to these settings [4, 5].

### Objectives

This review had three objectives. First, to identify measurement tools, instruments, and frameworks developed for or utilized in LMIC health systems and distill the specific constructs, indicators, and measurement approaches that accompany them. Second, to compare measurement approaches across integration type. Third, to identify gaps in measurement approaches and their validation. The intended outcomes are to catalogue available approaches to measuring integration that can be adapted by researchers and practitioners in LMICs, and to identify where further evidence is required.

## Methods

We adopted Arksey and O’Malley’s six stages for scoping reviews [15] and followed the checklist of Preferred Reporting Items for Systematic Reviews and Meta-Analyses’ Extension for Scoping Reviews (PRISMA-ScR) provided in Supplement 1 [19, 20].

### Conceptual framework: the rainbow model for integrated care

A modified version of the Rainbow Model for Integrated Care (RMIC) conceptual framework, first developed and utilized by Bautista et al. [13], was selected to guide the review and align findings to previous work. The RMIC is a conceptual framework for understanding integrated care through the lenses of primary health care (PHC) and people-centeredness. RMIC measurement tools have been validated in multiple settings [21–23], making the framework appropriate to guide a measurement-focused scoping review.

The framework delineates six types of integration [24], which we used to organize our analysis. At the macro-level, systems integration – either horizontally linking care across similar levels of the system or vertically linking different levels of care – requires a consistent set of formal or informal rules to ensure continuum of care [24]. At the meso-level, professional and organizational integration consider how care is delivered [25]. Organizational integration focuses on inter-organizational relationships and governance, while professional integration emphasizes inter-professional partnerships [24, 25]. Clinical integration exists at the micro-level and focuses on care coordination [24, 25]. Finally, normative and functional integration can occur at the micro-, meso-, and macro-level [24, 25]. Normative integration considers the existence of a shared mission, values, and culture, while functional integration emphasizes non-clinical services including financing, data systems, and management [24, 25].

### Search strategy

A replicable search strategy was developed using previous reviews on integration as index articles to select and refine search terms [12–14, 26]. Development of the search strategy was iterative and built upon the search strategy developed by Bautista et al. to promote comparability [13]. The starting search date of 15 June 2014 was selected to limit the overlap between this review period and past publications, due to an uptick in publications beginning in this period. The end date for the search was 21 November 2021 (the date of the final search). The final search strategy included four concepts: (i) integration and related constructs; (ii) instruments; (iii) measurement; and (iv) LMICs.

The search strategy was tested and refined in PubMed and translated for Embase and Web of Science. Supplement 2 includes the search results and search strategy for PubMed. Titles and abstracts were imported into Covidence systematic review software [27], and the duplicates were removed.

### Study screening and selection

Titles and abstracts were screened utilizing pre-determined inclusion and exclusion criteria described in Table 1. Like past reviews on integration, this review focuses on the process of integration, and we excluded papers focused solely on measuring integration outcomes [12, 13]. Unlike past reviews and for comprehensiveness, we included qualitative studies if they utilized an explicit framework, model, or theory to assess dimensions of integration. Given the diversity in terminology, we considered a paper eligible if aligned with the broad definition of integration developed by the World Health Organization (WHO) [28] and utilized either ‘integration’ or related terminology.

**Table 1:** Inclusion and exclusion criteria

Theme	Inclusion	Exclusion
*Geography*^a^	• Developed for any LMIC health system and/or for LMIC countries as a whole *OR* • Has been tested, validated, and/or piloted in any LMIC health system	• Developed for a HIC health system and used exclusively in that setting
*Study Content*	• Includes a specific framework, measurement instrument, or assessment approach for integration	• No framework or measurement instrument or assessment approach for integration is included in the article
*Scope of measurement instrument*	• Broadly aligns with the World Health Organization’s definition of integration: “The organization and management of health services so that people get the care they need, when they need it, in ways that are user friendly, achieve the desired results and provide value for money” [28] • Must intend to measure or assess one or more constructs related to integration (e.g. care continuity, care coordination) • Must be relevant to service delivery within the health sector • Must focus on the structure or process of integration, including planning for integration, implementing integration, experiences directly with the integration model, or evaluating integration as an intermediate output or outcome	• Does not measure constructs related to integration • Does not include dimensions of service delivery • Multi-sectoral instruments that are used exclusively outside the health sector • Focused solely on integration’s clinical outcomes or impact (for example, measuring reduced admissions or mortality changes because of integration) • Focused solely on patient experiences or preferences for integration outcomes
*Timeframe*	• Published between 15 June 2014 and 24 November 2021	• Published outside the inclusion period
*Language*	• Published in English • Available English translation	• Published in a language other than English with no translation available
*Type of study*	• Original research • Validation or pilot study of an instrument • Review article	• Commentary or editorials

^a^LMIC classification was determined by the World Bank’s Lending Classification Groups [29]

Two reviewers independently screened each title and abstract against the inclusion and exclusion criteria. Subsequently, the full text of each article was reviewed by three reviewers independently. All reviewers screened and charted a common set of index articles to promote consistency. When it was unclear whether to include or exclude an article per our criteria, the article was discussed by the entire research team against the review framework. If consensus was not reached, a senior member of the research team made the final determination.

### Data charting and extraction

Data was charted per a standardized extraction form developed specifically for this review in Microsoft Excel [30]. The extraction form was organized by the review’s conceptual framework and is provided in Supplement 3. The objective of the review was to capture and synthesize all available evidence within an evolving field, so we did not review articles for quality. Results were analyzed by the type of integration per the RMIC, as this was determined to be the most salient aspect of the framework.

### Patient and public involvement

None.

## Results

**Fig. 1:**
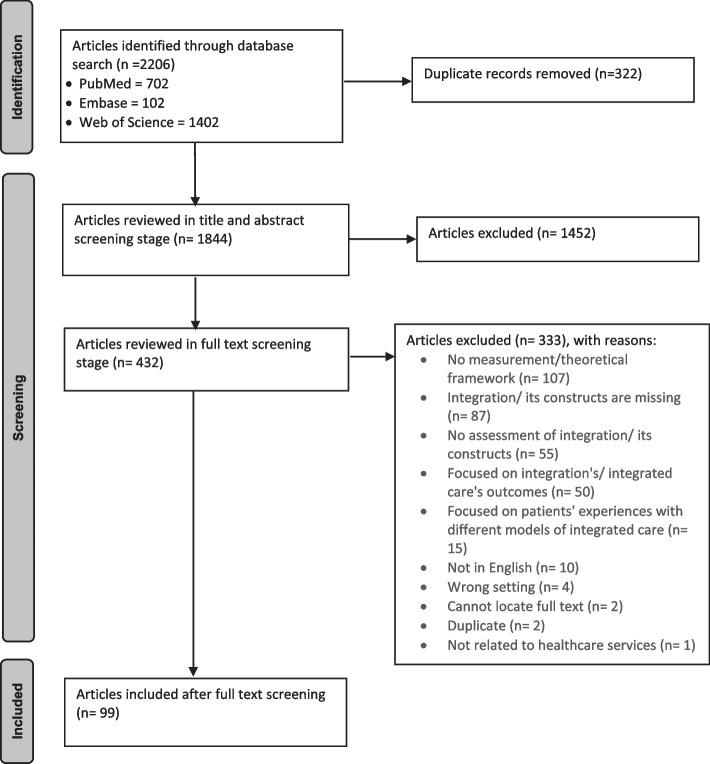
Preferred reporting items for systematic reviews and meta-analyses flow chart

A total of 2,206 studies were located across databases. 322 duplicates were excluded. Titles and abstracts of the remaining 1,884 articles were reviewed. 432 articles were included for full text review and simultaneous data extraction. Data extraction was conducted using 99 articles (Fig. 1).

### Study characteristics

Table 2 provides an overview of study characteristics for included studies, organized by the RMIC’s integration type.

**Table 2:** Study characteristics

	**Clinical**	**Organizational**	**Professional**	**Functional**	**Systems**	**Multiple**^a^	**Unable to determine or N/A**	**Total number of eligible articles** (*N* = 99)
**Publication Year**								
2014	2	1	1	0	1	2	1	8
2015–2016	7	2	0	0	3	8	1	21
2017–2018	12	3	0	0	0	6	2	22
2019–2020	14	4	3	0	0	9	1	33
2021	7	0	2	1	0	3	2	15
**Region, by World Bank Classification** [31]								
Global	0	0	0	0	0	0	2	2
Sub-Saharan Africa	37	1	1	1	3	7	1	51
East Asia and Pacific	1	6	2	0	0	6	0	15
Europe and Central Asia	0	0	2	0	0	0	0	2
Latin America and the Caribbean	1	2	0	0	1	14	4	22
Middle East and North Africa	0	1	1	0	0	1	0	3
South Asia	3	0	0	0	0	0	0	3
Multiple	0	0	0	0	0	1	0	1
**Service(s)**^b^								
HIV and others	31	1	1	0	1	7	0	41
MNCH and others	9	0	0	1	0	1	0	11
Mental health and others	0	0	0	0	0	3	0	3
PHC and others	0	2	1	0	1	12	1	17
Other NCD and others	2	3	0	0	0	4	0	9
Other	0	4	4	0	2	0	3	13
Not reported/ not applicable	0	0	0	0	0	2	3	5
**Study design**								
Quantitative	24	9	6	0	2	14	5	60
Qualitative	5	0	0	1	0	2	2	10
Mixed or multi-methods	13	1	0	0	2	13	0	29
**Study population**								
Patients	12	3	0	0	0	3	3	21
Providers	14	3	5	1	1	11	0	35
Government	0	0	0	0	0	1	0	1
Multiple	9	2	1	0	1	9	1	23
Not reported/ not applicable	7	2	0	0	2	5	3	19
**Country income level, by World Bank Classification** [31]								
Upper middle income	7	8	3	0	1	21	4	44
Lower-middle income	21	1	2	0	2	5	0	31
Low income	11	1	1	1	1	1	1	17
Multiple income levels	3	0	0	0	0	2	1	6
Not reported/ not applicable	0	0	0	0	0	0	1	1

^a^Multiple refers to studies that had more than one type of integration present in the study

^b^Abbrev: *HIV* Human Immune Deficiency Virus, *MNCH* Maternal, newborn, and child health, *PHC* Primary Health Care, *NCDs* Non-communicable diseases. The service listed was deemed the most prominent service identified in the paper, or the service that another service was being integrated into. For example, integration of hypertension screening into an HIV clinic would be categorized as “HIV and others” while integration of HIV counseling into an existing hypertension screening program would be considered “NCD and others”

#### Study population

The most common study populations in our sample were health care providers, followed by patients. Studies that included multiple participants included government, non-governmental organizations, health managers, researchers, donors, and communities.

#### Geography and income level

**Fig. 2:**
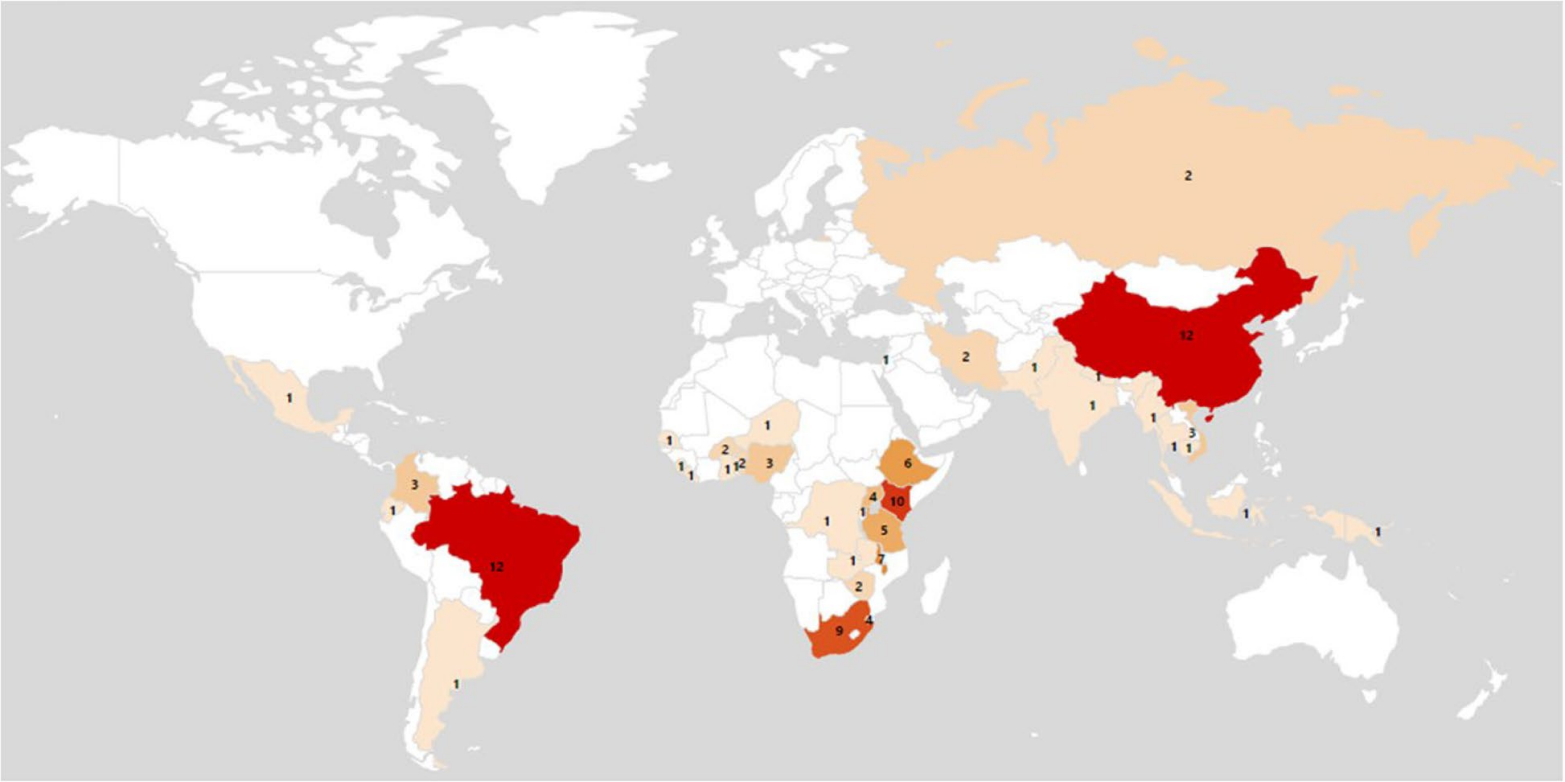
The number of included studies, by country, in the final sample of included papers

Our inclusion criteria included any LMIC; however, included studies clustered within specific income levels, regions, and countries (Fig. 2). Middle-income countries were more represented (*n* = 74) than low-income countries (*n* = 17). Within sub-Saharan Africa, 70.58% of papers were from five countries (Kenya, *n* = 10; South Africa, *n* = 8; Ethiopia, Swaziland, Malawi, *n* = 6). 45.45% of papers from Latin America and Caribbean were from Brazil, and 75% of papers from East Asia and Pacific were from China.

#### Types of services

Included studies were largely focused on a few health services. HIV services were commonly integrated with sexual and reproductive health (SRH) care (*n* = 9), family planning (FP) (*n* = 7), TB (*n* = 4) and non-communicable diseases (NCDs) (*n* = 4). PHC services, both alone (*n* = 11) and vertically integrated with secondary health care (*n* = 4), were the second most common.

#### Approaches to assessing integration

Table 3 maps included articles against each component of the review framework. We present the results by the type of integration as defined by the RMIC and identify commonly utilized constructs, indicators, and methods for each integration type. Supplement Three provides the full data extraction sheet.

**Table 3:** Categorizing included papers along the components of the review framework

	**Clinical**	**Organizational**	**Professional**	**Functional**	**Systems**	**Multiple**	**Total number of eligible articles** (*N* = 99)^b^
**Type of integration**^a^	42	10	6	1	4	29	**92**
Not Applicable/ unable to determine							**7**
**Level of integration (*n* = 92)**							
Micro	28	0	0	0	0	0	**28**
Meso	9	10	6	0	0	12	**37**
Macro	0	0	0	1	4	2	**7**
Multiple	2	0	0	0	0	13	**15**
Not Applicable / unable to determine	3	0	0	0	0	2	**5**
**Focus of integration (*n* = 92)**							
Population	38	10	6	1	4	22	**81**
Person	0	0	0	0	0	0	**0**
Both	1	0	0	0	0	2	**3**
Not Applicable/ unable to determine	3	0	0	0	0	5	**8**
**Continuum of integration (*n* = 92)**							
Full	2	2	0	0	0	5	**9**
Coordination	1	4	1	0	1	1	**8**
Linkage	4	0	0	0	0	3	**7**
Multiple	1	3	0	0	2	6	**12**
Not Applicable/ unable to determine	34	1	5	1	1	14	**56**
**Continuum of care (*n* = 92)**							
Health Promotion	1	0	0	0	0	1	**2**
Disease Prevention	3	0	0	0	0	1	**4**
Diagnosis and Treatment	20	1	1	0	0	5	**27**
Rehabilitation	0	0	0	0	0	0	**0**
Long-term and Palliative	0	0	0	0	0	0	**0**
Multiple	12	6	2	1	1	10	**32**
Not Applicable/ unable to determine	6	3	3	0	3	12	**27**
**Level of service delivery (*n* = 92)**							
Community	1	0	1	1	0	0	**3**
Primary (Facility)	14	2	1	0	1	7	**25**
Secondary	2	0	0	0	0	0	**2**
Tertiary	0	0	0	0	0	0	**0**
Multiple	14	6	2	0	3	16	**41**
Not Applicable/ unable to determine	11	2	2	0	0	6	**21**

^a^No papers measured normative integration alone, so it is not represented in the table columns

^b^The total number of included papers is 99; seven papers could not be categorized clearly by integration type, therefore the total number of articles categorized by the other categories is 92

### Clinical integration (micro-level)^1^

The RMIC defines clinical integration as, “the coordination of person-focused care in a single process across time, place, and discipline.” [25] 42 papers were determined to measure clinical integration alone, making it the most common type of integration measured by included studies.

#### Quantitative methods used to measure clinical integration

We identified some consistency in the indicators utilized for clinical integration. Most often, indicators for two or more services were developed specifically for the study and measured the extent to which those services were delivered jointly. The two most common indicators were variations on (1) testing, screening, or counseling a patient with one condition for the other condition or service need [32–46]; and (2) availability of services on the same day, whether via joint treatment by the same provider or in the same visit by different providers [38, 40, 41, 46–58].

Other indicators included variations on (3) internal and external referral practices [32, 41, 42, 47]; (4) common treatment or counseling spaces [41, 47, 50]; (5) availability or receipt of commodities, equipment, and or medications for two or more conditions [38, 44, 49, 50, 52, 59]; and (6) provider knowledge on two or more services [35, 39, 52, 55, 60].

#### Surveys to measure clinical integration

Eighteen papers developed or adapted survey instruments to measure clinical integration; of these, 10 papers surveyed health care providers [34, 35, 38, 40, 42, 46, 60–63], and eight surveyed patients [35, 43, 44, 48, 54, 59, 62, 64].

Papers measuring clinical integration often combined provider survey data with other methods to assess the extent of integration. For example, Pfaff et al*.* developed an integration indicator for the availability of NCD and anti-retroviral therapy (ART) services, which was accompanied by a series of facility readiness indicators and qualitative interviews to assess the ability of HIV programs to provide NCD care [38]. Sheahan et al*.* utilized provider surveys and facility audit data to develop a provider and facility integration index, which was analyzed longitudinally to understand the extent of integration in comparison to baseline facility and provider characteristics [46]. Similarly, Milford et al*.* conducted baseline and endline provider surveys to measure whether service integration improved after an HIV and SRH service integration training intervention [42].

Papers administering patient surveys focused on similar indicators but relied on patient experience or recall of how services were provided. For example, Church et al*.* and Biswas et al*.* used client exit surveys to assess integrated services and counseling received during patient visits [43, 64].

#### Use of secondary data sources

Fifteen papers relied on secondary data to measure clinical integration. Of these, 10 used facility-level data [32, 34, 35, 49, 50, 52, 55, 57, 58, 65], and five used patient medical records [33, 37, 39, 59, 66].

At the facility-level, Adamchak et al*.* determined the feasibility of measuring the integration of family planning (FP) and HIV services from existing health management information systems (HMIS) [32]. HMIS indicators included the proportion of HIV-related service clients screened for FP and the proportion of FP clients that received an HIV test at a family planning service delivery point. Kanyangarara et al*.* used data from existing facility surveys to measure the availability of integrated FP services in HIV treatment facilities [52].

Patient data included medical records and household surveys. Using patient records, Moucheraud et al*.* examined whether patients filled hypertension medication and ART during a single visit [59], while Mitiku et al*.* assessed the proportion of TB patients tested for HIV and vice versa [33].

#### Other quantitative methods

Four papers used economic analysis to compare the costs of integrated service models compared to non-integrated models [67–70]. We considered cost-effectiveness as a plausible approach for assessing the level of integration and therefore included it in the review. For example, Wall et al*.* compared the actual costs of an integrated HIV and FP program to the historical costs of providing separate services [67], and Vodicka et al*.* estimated costs using time in motion data for integrating cervical cancer screening into HIV care [68]. Finally, three papers used a facility audit checklist or protocol to assess how integrated services were provided [41, 46, 47].

#### Qualitative methods to measure clinical integration

Eleven papers used interviews to assess clinical integration [39–41, 45, 55, 62, 64, 65, 71–73]. For example, Irungu conducted in-depth provider interviews to assess adherence with integrated service delivery protocols for pre-exposure prophylaxis (PrEP) implementation within HIV care [45].

Four papers used focus group discussions to assess barriers, enablers, and patient experiences with specific programs [36, 71, 72, 74]. Three papers utilized observations to assess how integrated services were provided at the health facility [41, 47, 50]. For example, McGinn et al*.* conducted facility audits in Malawi to observe HIV and FP services with a checklist to assess whether integrated treatment guidelines were applied at the facility. Qualitative data was then transformed into a quantitative percentage score [41]. Finally, two papers used client flow analysis to track how patients moved through clinical services [56, 74].

### Organizational integration (meso-level)

Organizational integration is defined by the RMIC as, “inter-organizational relationships […] including common governance mechanisms, to deliver comprehensive services to a defined population.” [25] 10 papers were categorized as measuring organizational integration alone [75–84]. Organizational integration measurement constructs focused on inter-organizational coordination of care and collaboration.

#### Quantitative and qualitative methods to measure organizational integration

Of the 10 papers measuring organizational integration, seven utilized surveys to measure organizational integration [77–83]. For example, Li et al*.* adapted a measurement instrument to measure governance, shared goals and vision, formalization and internalization across collaborating health care organization providing NCD care [77]. Thomas et al. applied a network density analysis to measure the density of organizational networks providing integrated HIV and FP services, comparing the links, density, centralization, and reciprocity of organizations that received a network strengthening initiative compared to one without intervention [79].

Two papers used quantitative approaches to develop new or adapted organizational integration constructs in a specific health system [76, 84]. For example, Seyedani et al*.* developed an adapted model for organizational integration for the Iranian health system [84]. Factor analysis derived five organizational integration aspects: learning organizations, inter-organizational strategies, organizational features, interest management, and coordination of care and information [84].

Finally, Wang et al*.* applied a mixed method approach [75]. Three indices were used to measure the effect of organizational integration on care coordination compared to best practices [75].

### Professional integration (meso-level)

Professional integration is defined as, “inter-professional partnerships based on shared competences, roles, responsibilities and accountability to deliver a comprehensive continuum of care to a defined population” in the RMIC [25]. Six papers were deemed to measure professional integration alone [85–90]. These papers emphasize measurement constructs of communication and collaboration across providers, leadership, and collaboration.

#### Quantitative and qualitative methods to measure professional integration

All papers measuring professional integration were quantitative and utilized surveys of providers [85–88, 90]; one paper also included government officials and patients in the study population [89]. Constructs or indicators used to measure professional integration were similar across included papers and emphasized communication across medical professionals for referrals or treatment across levels of the health system [86, 89], leadership and decision making [88], teamwork [85–87], provider communication for coordination of care [87–89], and provider collaborative capacity [90]. For example, Li et al*.* measured the change in professional integration within an integrated community HIV and addiction program [85]. Providers were randomized to receive a training on HIV and addiction services, and baseline and endline surveys were administered to providers to measure the relative increase in provider collaboration across the treatment and control groups [85]. Tao et al*.* assessed professional integration by calculating coordination scores for patients and providers [89]. Similarly, Shaqura et al*.* developed a new tool to measure interprofessional collaboration, with survey items focused on leadership, mission, communication, coordination, and decision making [88].

### Systems integration (macro-level)

The RMIC defines systems integration as, “a horizontal and vertically integrated system, based on a coherent set of (formal and informal) rules and policies between care providers and external stakeholders for the benefit of people and populations.” [25]. Four papers measured systems integration [91–94], generally focused on the presence of specific services across health system building blocks [95].

#### Quantitative and qualitative methods to measure systems integration

Two papers utilized quantitative tools to measure vertical [94] or horizontal [93] systems integration. For example, Mbah et al*.* developed a facility-level integration checklist, which measured the extent of integration between HIV and general laboratory services [93]. Indicators of integration included common training, equipment, standard operating procedures, physical location, and quality assurance processes [93].

Two papers employed mixed methods approaches. Deconinck et al*.* measured the integration of acute malnutrition interventions into health systems using 29 indicators [92] aligned with the WHO’s six health systems building blocks [95]. Mensa et al*.* collected data on the extent of integration of neglected tropical diseases into the heath system through qualitative interviews organized around health systems components [91]. Qualitative data was converted to an index with scoring at three levels of the health system [91].

### Functional integration (micro-, meso-, and macro-levels)

Per the RMIC, functional integration focuses on support functions, such as financial, management, and information systems [25]. One paper was categorized as measuring functional integration alone [96]. Mossie (2021) assessed the use of a common medical record card for use across the maternal health and FP service continuum at the PHC (community) level [96]. The authors used interviews to understand whether common medical record card was utilized to improve coordination and continuity of care [96].

### Normative integration (micro-, meso-, and macro-levels)

According to the RMIC, normative integration is, “the development and maintenance of a common frame of reference” [25]. Normative integration was considered in four papers alongside other types of integration [97–100], and focused on shared values, culture, and vision. For example, Xu assessed the ‘degree of sharing of organizational culture’ and ‘adherence to public health service goal’ across three different integration models [100].

### Multiple types of integration

We considered a study as measuring multiple types of integration if the indicators or framework components cut across multiple integration types as defined by the RMIC. 29 papers were categorized as measuring multiple types of integration.

Quantitative and qualitative methods to measure multiple types of integration in a single study.

Of the 29 studies measuring multiple types of integration, clinical integration (*n* = 19) and professional integration (*n* = 19) were the most common, followed by systems (*n* = 15), organizational (*n* = 13), functional (*n* = 9), and normative (*n* = 4). The most common combinations identified were clinical and professional (*n* = 4) and clinical and systems (*n* = 4).

#### Quantitative methods to measuring multiple types of integration

Eighteen papers describe a survey or questionnaire; of those, providers were the most surveyed (*n* = 14) [98–111]. Tang et al*.* developed a survey tool to measure cooperative behavior of physicians across individual and organizational factors, which included a range of items from culture and leadership to training and referrals [99].

Patients (*n* = 6) [98, 103, 106, 112–114], and health care managers (*n* = 3) were also surveyed [103, 110, 115]. For example, De Almedia et al*.* surveyed patients about access to care, scheduling, satisfaction, and awareness, while health providers and managers were asked about care provision, referral instruments, medical records, and protocols [106].

Secondary data was utilized in four studies [23, 116, 117]. Miguel-Esponda et al*.* used patient records to determine fidelity to integration protocols, and interviews were conducted to assess the extent of penetration of mental health care into PHC [116].

Other methods included facility audits [97, 118] and social network analysis [119]. Afrizal et al*.* assessed different integration types using quantitative data from ANC registers and qualitative interview data from midwives implementing an integrated ANC scheme [97]. Van Rensburg et al*.* combined semi-structured interviews with social network analysis to assess the nature and extent of collaborative relationships between state and non-state service providers [119].

#### Qualitative methods to measuring multiple types of integration

For qualitative methods, nine papers conducted semi-structured interviews; providers were the most common participants (*n* = 8) [97, 99, 100, 102, 106, 113, 118, 120], followed by health care managers (*n* = 4) [97, 113, 118, 120], and patients (*n* = 2) [100, 113].

Other qualitative methods included patient journey mapping [115] and facility-level observations [110, 118]. Bousquat et al*.* combined patient journey mapping with a provider and health manager survey [115]. Constructs measured included administrative and organizational structures, organization, team service integration, and information continuity [115]. An accompanying therapeutic itineraries approach mapped patient journeys against continuity of care components [115].

## Discussion

Through this review, we identified 99 studies measuring integration in the health care sector in LMICs. The multitude of frameworks and constructs used by included studies illustrates the complexity and contextual nature of integration. Our review supports the assertion that integration suffers from conceptual immaturity [121] with inconsistent terminology and definitions serving as barriers to consolidate and compare findings.

### Comparing measurement approaches identified

Analyzing included studies by the review framework identified patterns in measurement approaches. Similar to past reviews [13], the largest number of papers in our sample were measuring clinical integration. Papers measuring clinical integration were more likely to incorporate patients as a study population and were more likely to utilize existing secondary data. Indicators prioritized the integration of two or more services into a ‘one-stop-shop’ or point of care model. Professional and organizational integration papers were more likely to utilize provider surveys and were the most likely to be categorized along the continuum of integration. Professional integration constructs emphasized teamwork and collaboration across providers, while organizational integration measured similar constructs across organizations. Papers focused on systems integration represented a range of constructs and measurement approaches that were specifically tailored to the context. Indicators for functional integration focused on the integration of information systems. Constructs for normative integration focused largely on shared value systems and were only provided in tandem with other types of integration.

### Gaps identified

#### Concentration of evidence in specific services and geographies

While our review included all LMICs, 58.65% of papers came from seven countries (China, Brazil, Kenya, South Africa, Ethiopia, Swaziland, Malawi), and 41.41% of papers measured the integration of another service into HIV care. Despite recommendations to consider integration across the continuum of care, nearly all studies focused on curative services, and no papers measured the integration of physical rehabilitation or palliative services. A key takeaway is that the focus of integration efforts and the resulting measurement approaches are context-specific, both in terms of which conditions or services are integrated and in the broader health systems structures responsible for that integration. However, normative recommendations for integration are often defined as service or context neutral. The mismatch between context-specific integration evidence and broader normative recommendation warrants further empirical inquiry.

#### Emphasis on specific integration models

Categorizing measurement approaches by the RMIC’s integration types provided insights into the goals of integration models. Indicators for clinical integration were often the single focus of a study, designed for integrating vertical disease programs (especially HIV) and focused on increasing service accessibility and utilization. In contrast, all other integration types were more likely to be measured in combination. This reflected more expansive horizontal and vertical integration models across the continuum of care. Like past reviews, our review identified limited measurement approaches for functional, systems, and normative integration [13].

Previous work indicates that integration models in LMICs are more likely to focus on the integration of two or more services to improve access or uptake [5]. Our findings from the Africa region reflected this, with papers largely focused on clinical integration of HIV and other services. In contrast, papers from Latin America and Asia more frequently reflected professional and organizational integration types more commonly associated with HICs [5]. 77% of included studies in Bautista et al.’s 2017 review on integration came from HIC, the majority of which focused on clinical integration, highlighting variation of integration models across country income levels [13]. Additional research can identify which integration models are common across different regions and service types.

Integration of entire sectors or service categories into the health system is especially poorly researched and likely to be particularly complex. Only two studies in our review measured a whole-sector approach to integration (examining nutrition and neglected tropical diseases) and highlight the importance of developing indicators across levels and components of the health system [91, 92]. These examples may be particularly instructive for the recent calls to integrate rehabilitation into health systems [16] and emphasize the importance of measuring non-clinical components to integration.

#### The need for publicly available instruments

Compared to past reviews [13], few studies in our review reported validity or reliability measures for quantitative instrument development, and qualitative studies rarely reported trustworthiness (Supplement 3). Few tools were publicly available, challenging the ability of future researchers to build on and adapt approaches we have documented here. In other cases, methodologies – for example, qualitative interview data converted to quantitative integration scores – lacked adequate description for outside readers, rendering the methods of measurement irreplicable. Therefore, while the findings of this review provide ideas for future researchers, it points to the need for appropriate documentation of measurement methods and availability of tools for adaptation.

Understanding implementation challenges for these tools and shared learnings are also crucial to develop consensus around utility of the above-mentioned tools and approaches. This would help to reduce redundancy, support the development of a comparable evidence-base for comprehensive measurement of integration across levels of the health system, and facilitate adaptation of tools to make them relevant for specific contexts and services.

### Strengths and limitations

Our broad definition of measurement was a strength. This allowed us to capture qualitative and mixed methods articles that assessed integration through perception or experiences of target groups, as well as novel methodologies such as social network analysis. The exclusion of non-English papers was a limitation and resulted in the exclusion of ten papers.

The use of the adapted RMIC as the basis of the review framework was both a strength and limitation. Despite the original focus of the RMIC on PHC, the integration types present in the RMIC were identifiable in most included papers. This allowed us to use an organizing heuristic for the review based on existing literature and provided a theory-based approach to knowledge synthesis on a fragmented topic. However, included papers often utilized different definitions or terminology, which made it challenging to categorize. Few papers reported sufficient detail to be categorized across all the review framework’s components.

Some papers aligned with broader definitions or models of integration put forward by the WHO rather than aligning clearly to the definition of integrated care for the RMIC. We included these papers in our review as they fit our inclusion criteria and provided insights into measurement approaches. However, categorizing these approaches against the RMIC required additional interpretation of both the original framework and included papers.

A related observation is that many papers from LMICs were assessing new models for integration and were operational in nature, while the original RMIC framework is conceptual. This manifested in the way included studies were organized, often defining measurement constructs by components of an integration model that cut across multiple integration types. For example, we categorized measurement of the referral pathway to promote care continuity differently depending on the wording of survey items – provider collaboration was categorized as professional integration, intra-organizational partnerships were categorized as organizational integration, macro-level referral policies were categorized as systems integration, and common information management system to manage referrals was categorized as functional integration. Therefore, similar studies seeking to measure care continuity through referrals could be categorized differently based on the specific measurement approach. This is a limitation, both of our review and the broader literature.

Finally, our search began in June 2014 to align with past reviews; however, previous reviews were limited to quantitative studies. There may be additional qualitative studies measuring integration prior to the start of our search which are not captured in an existing review on this topic.

## Conclusion

This review aimed to categorize and describe measurement approaches for integration in LMICs. Our findings describe measurement approaches that can be adapted for future research and practice and identify critical conceptual and practical gaps towards strengthening measurement of integration in LMIC health systems.

### Supplementary Information

**Additional file 1.** Preferred Reporting Items for Systematic reviews and Meta-Analyses extension for Scoping Reviews (PRISMA-ScR) Checklist.

**Additional file 2.** List of databases and results of search. PubMed Search Strategy.

**Additional file 3.** Data Extraction Sheet for Integration measurement and its applications in LMIC country health systems: a scoping review.

## Data Availability

All data analyzed in this study is provided in Supplement 3. This includes the data extraction sheet and complete list of included papers.

## Footnotes

1 In this and the following results sections, the number of papers is reported per method utilized. Some papers used multiple methods and are counted more than once.
