# Supplementary material for: Integration measurement and its applications in low- and middle-income country health systems: a scoping review
Source: BMC Public Health. 2023 Sep 28;23:1876. doi: 10.1186/s12889-023-16724-2 (PMC10537146; doi:10.1186/s12889-023-16724-2)
Supplement: Supplementary file 2 — Additional file 2. List of databases and results of search. PubMed Search Strategy. [file 12889_2023_16724_MOESM2_ESM.docx]

**Supplement Two**

**List of databases and results of search**

|  | **Database Name** | **Link** | **April 9, 2021, search results** | **November 24, 2021, search results^1^** |
| --- | --- | --- | --- | --- |
| 1 | *PubMed* | <https://pubmed.ncbi.nlm.nih.gov/> | 625 | 77 |
| 2 | *Embase* | <https://www.embase.com/login#search> | 90 | 12 |
| 3 | *Web of Science* | [www.webofknowledge.com](http://www.webofknowledge.com) | 1,268 | 134 |
|  | ***Total results*** |  | ***1,983*** | ***223*** |

^1^ *Following the initial data extraction, the search strategy was re-run to ensure up-to-date findings of the review and articles published between April and November 2021 were incorporated.*

**PubMed Search Strategy**

**Concept: Integrated health service delivery (modified from Bautista et al.)**[1]

(“coordination of care”[tiab] OR “care coordination”[tiab] OR “Healthcare coordination”[tiab] OR “health care coordination”[tiab] OR “Service coordination”[tiab] OR “Services coordination”[tiab] OR “Service coordination”[tiab] OR “Coordinated care”[tiab] OR “Coordinated health care”[tiab] OR “Coordinated healthcare”[tiab] OR “Coordinated patient care”[tiab] OR “Coordinated service”[tiab] OR “Coordinated services”[tiab] OR “Coordinated care”[tiab] “Co-ordinated service”[tiab] OR “Co-ordinated services”[tiab] OR “Coordinating care”[tiab] OR “Coordinating health care”[tiab] OR “Coordinating patient care”[tiab] OR “Coordinating service”[tiab] OR “Coordinating services”[tiab] OR “Coordinating care”[tiab] OR “Co-ordinating health care”[tiab] OR “Co-ordinating service”[tiab] OR “Co-ordinating services”[tiab]))) OR ((((“Delivery of Health Care, Integrated” [MeSH]) OR (“Integration of care”[tiab] OR “Integration of health care”[tiab] OR “Integration of healthcare”[tiab] OR “Integration of patient care”[tiab] OR “Integration of service”[tiab] OR “Integration of services”[tiab] OR “Care integration”[tiab] OR “Healthcare integration”[tiab] OR ”health care integration”[tiab] OR “Service integration”[tiab] OR “Services integration”[tiab] OR “Integrated care”[tiab] OR “Integrated health care”[tiab] OR “Integrated healthcare”[tiab] OR “Integrated patient care”[tiab] OR “Integrated service”[tiab] OR “Integrated services”[tiab] OR “Integrating care”[tiab] OR “Integrating health care”[tiab] OR “Integrating healthcare”[tiab] OR “Integrating patient care”[tiab] OR “Integrating service”[tiab] OR “Integrating services”[tiab] OR “health care system integration”[tiab] OR “health system integration”[tiab] OR “horizontal integration”[tiab] OR “vertical integration”[tiab] OR “organizational integration”[tiab] OR “professional integration”[tiab] OR “clinical integration”[tiab] OR "Continuity of patient care"[tiab] OR "Continuity of care"[tiab] OR "patient care continuity"[tiab] OR "care continuity"[tiab] OR "health care continuity"[tiab] OR "service continuity"[tiab])))

**Concept: Instruments (taken directly from Bautista et al.)**[1]

(((((((("surveys and questionnaires"[Mesh]) OR questionnaire*[tiab]) OR assess*[tiab]) OR measure*[tiab]) OR survey*[tiab]) OR test[tiab]) OR "survey instrument"[tiab]))

*the mesh terms were updated to the current version in PubMed from Bautista

**Concept: Measurement (taken directly from Bautista et al.)**[1]

(((("Validation Studies as Topic" [MeSH]) OR ((instrumentation[sh] OR methods[sh] OR Validation Studies[pt] OR Comparative Study[pt] OR “psychometrics”[MeSH] OR psychometr*[tiab] OR clinimetr*[tw] OR clinometr*[tw] OR “observer variation”[MeSH] OR observer variation[tiab] OR “Delivery of Health Care, Integrated”[Mesh] OR “reproducibility of results”[MeSH] OR reproducib*[tiab] OR “discriminant analysis”[MeSH] OR reliab*[tiab] OR unreliab*[tiab] OR valid*[tiab] OR coefficient[tiab] OR homogeneity[tiab] OR homogeneous[tiab] OR “internal consistency”[tiab] OR (cronbach*[tiab] AND (alpha[tiab] OR alphas[tiab])) OR (item[tiab] AND (correlation*[tiab] OR selection*[tiab] OR reduction*[tiab])) OR agreement[tiab] OR precision[tiab] OR imprecision[tiab] OR “precise values”[tiab] OR test–retest[tiab] OR (test[tiab] AND retest[tiab]) OR (reliab*[tiab] AND (test[tiab] OR retest[tiab])) OR stability[tiab] OR interrater[tiab] OR inter-rater[tiab] OR intrarater[tiab] OR intra-rater[tiab] OR intertester[tiab] OR inter-tester[tiab] OR intratester[tiab] OR intra-tester[tiab] OR interobserver[tiab] OR inter-observer[tiab] OR intraobserver[tiab] OR intraobserver[tiab] OR intertechnician[tiab] OR inter-technician[tiab] OR intratechnician[tiab] OR intra-technician[tiab] OR interexaminer[tiab] OR interexaminer[tiab] OR intraexaminer[tiab] OR intra-examiner[tiab] OR interassay[tiab] OR inter-assay[tiab] OR intraassay[tiab] OR intra-assay[tiab] OR interindividual[tiab] OR inter-individual[tiab] OR intraindividual[tiab] OR intra-individual[tiab] OR interparticipant[tiab] OR interparticipant[tiab] OR intraparticipant[tiab] OR intra-participant[tiab] OR kappa[tiab] OR kappa’s[tiab] OR kappas[tiab] OR repeatab*[tiab] OR ((replicab*[tiab] OR repeated[tiab]) AND (measure[tiab] OR measures[tiab] OR findings[tiab] OR result[tiab] OR results[tiab] OR test[tiab] OR tests[tiab])) OR generaliza*[tiab] OR generalisa*[tiab] OR concordance[tiab] OR (intraclass[tiab] AND correlation*[tiab]) OR discriminative[tiab] OR “known group”[tiab] OR factor analysis[tiab] OR factor analyses[tiab] OR dimension*[tiab] OR subscale*[tiab] OR (multitrait[tiab] AND scaling[tiab] AND (analysis[tiab] OR analyses[tiab])) OR item discriminant[tiab] OR interscale correlation*[tiab] OR error[tiab] OR errors[tiab] OR “individual variability”[tiab] OR (variability[tiab] AND (analysis[tiab] OR values[tiab])) OR (uncertainty[tiab] AND (measurement[tiab] OR measuring[tiab])) OR “standard error of measurement”[tiab] OR sensitiv*[tiab] OR responsive*[tiab] OR ((minimal[tiab] OR minimally[tiab] OR clinical[tiab] OR clinically[tiab]) AND (important[tiab] OR significant[tiab] OR detectable[tiab])AND(change[tiab] OR difference[tiab])) OR (small*[tiab] AND (real[tiab] OR detectable[tiab]) AND (change[tiab] OR difference[tiab])) OR meaningful change [tiab] OR “ceiling effect”[tiab] OR “floor effect”[tiab] OR “Item response model”[tiab] OR IRT[tiab] OR Rasch[tiab] OR “Differential item functioning”[tiab] OR DIF[tiab] OR “computer adaptive testing”[tiab] OR “item bank”[tiab] OR “cross-cultural equivalence”[tiab]))))

**Concept: Low- and middle-income countries**[2]

afghanistan[tw] OR albania[tw] OR algeria[tw] OR american samoa[tw] OR angola[tw] OR antigua[tw] OR barbuda[tw] OR argentina[tw] OR armenia[tw] OR armenian[tw] OR aruba[tw] OR azerbaijan[tw] OR bahrain[tw] OR bangladesh[tw] OR barbados[tw] OR belarus[tw] OR byelarus[tw] OR belorussia[tw] OR byelorussian[tw] OR belize[tw] OR british honduras[tw] OR benin[tw] OR dahomey[tw] OR bhutan[tw] OR bolivia[tw] OR bosnia[tw] OR herzegovina[tw] OR botswana[tw] OR bechuanaland[tw] OR brazil[tw] OR brasil[tw] OR bulgaria[tw] OR burkina faso[tw] OR burkina fasso[tw] OR upper volta[tw] OR burundi[tw] OR urundi[tw] OR cabo verde[tw] OR cape verde[tw] OR cambodia[tw] OR kampuchea[tw] OR khmer republic[tw] OR cameroon[tw] OR cameron[tw] OR cameroun[tw] OR central african republic[tw] OR ubangi shari[tw] OR chad[tw] OR chile[tw] OR china[tw] OR colombia[tw] OR comoros[tw] OR comoro islands[tw] OR mayotte[tw] OR congo[tw] OR zaire[tw] OR costa rica[tw] OR cote d'ivoire[tw] OR ivory coast[tw] OR croatia[tw] OR cuba[tw] OR cyprus[tw] OR czech republic[tw] OR czechoslovakia[tw] OR djibouti[tw] OR french somaliland[tw] OR dominica[tw] OR dominican republic[tw] OR ecuador[tw] OR egypt[tw] OR united arab republic[tw] OR el salvador[tw] OR equatorial guinea[tw] OR spanish guinea[tw] OR eritrea[tw] OR estonia[tw] OR eswatini[tw] OR swaziland[tw] OR ethiopia[tw] OR fiji[tw] OR gabon[tw] OR gabonese republic[tw] OR gambia[tw] OR georgia[tw] OR georgian[tw] OR ghana[tw] OR gold coast[tw] OR gibraltar[tw] OR greece[tw] OR grenada[tw] OR guam[tw] OR guatemala[tw] OR guinea[tw] OR guyana[tw] OR guiana[tw] OR haiti[tw] OR hispaniola[tw] OR honduras[tw] OR hungary[tw] OR india[tw] OR indonesia[tw] OR timor[tw] OR iran[tw] OR iraq[tw] OR isle of man[tw] OR jamaica[tw] OR jordan[tw] OR kazakhstan[tw] OR kazakh[tw] OR kenya[tw] OR kosovo[tw] OR kyrgyzstan[tw] OR kirghizia[tw] OR kirgizstan[tw] OR kyrgyz republic[tw] OR kirghiz[tw] OR laos[tw] OR lao pdr[tw] OR lao people's democratic republic[tw] OR latvia[tw] OR lebanon[tw] OR lesotho[tw] OR basutoland[tw] OR liberia[tw] OR libya[tw] OR libyan arab jamahiriya[tw] OR lithuania[tw] OR macau[tw] OR macao[tw] OR macedonia[tw] OR madagascar[tw] OR malagasy republic[tw] OR malawi[tw] OR nyasaland[tw] OR malaysia[tw] OR maldives[tw] OR indian ocean[tw] OR mali[tw] OR malta[tw] OR micronesia[tw] OR kiribati[tw] OR marshall islands[tw] OR nauru[tw] OR northern mariana islands[tw] OR palau[tw] OR tuvalu[tw] OR mauritania[tw] OR mauritius[tw] OR mexico[tw] OR moldova[tw] OR moldovian[tw] OR mongolia[tw] OR montenegro[tw] OR morocco[tw] OR ifni[tw] OR mozambique[tw] OR portuguese east africa[tw] OR myanmar[tw] OR burma[tw] OR namibia[tw] OR nepal[tw] OR netherlands antilles[tw] OR nicaragua[tw] OR niger[tw] OR nigeria[tw] OR oman[tw] OR muscat[tw] OR pakistan[tw] OR panama[tw] OR papua new guinea[tw] OR paraguay[tw] OR peru[tw] OR philippines[tw] OR philipines[tw] OR phillipines[tw] OR phillippines[tw] OR poland[tw] OR polish people's republic[tw] OR portugal[tw] OR portuguese republic[tw] OR puerto rico[tw] OR romania[tw] OR russia[tw] OR russian federation[tw] OR ussr[tw] OR soviet union[tw] OR union of soviet socialist republics[tw] OR rwanda[tw] OR ruanda[tw] OR samoa[tw] OR pacific islands[tw] OR polynesia[tw] OR samoan islands[tw] OR sao tome and principe[tw] OR saudi arabia[tw] OR senegal[tw] OR serbia[tw] OR seychelles[tw] OR sierra leone[tw] OR slovakia[tw] OR slovak republic[tw] OR slovenia[tw] OR melanesia[tw] OR solomon island[tw] OR solomon islands[tw] OR norfolk island[tw] OR somalia[tw] OR south africa[tw] OR south sudan[tw] OR sri lanka[tw] OR ceylon[tw] OR saint kitts and nevis[tw] OR st kitts and nevis[tw] OR saint lucia[tw] OR st lucia[tw] OR saint vincent[tw] OR st vincent[tw] OR grenadines[tw] OR sudan[tw] OR suriname[tw] OR surinam[tw] OR syria[tw] OR syrian arab republic[tw] OR tajikistan[tw] OR tadjikistan[tw] OR tadzhikistan[tw] OR tadzhik[tw] OR tanzania[tw] OR tanganyika[tw] OR thailand[tw] OR siam[tw] OR timor leste[tw] OR east timor[tw] OR togo[tw] OR togolese republic[tw] OR tonga[tw] OR trinidad[tw] OR tobago[tw] OR tunisia[tw] OR turkey[tw] OR turkmenistan[tw] OR turkmen[tw] OR uganda[tw] OR ukraine[tw] OR uruguay[tw] OR uzbekistan[tw] OR uzbek[tw] OR vanuatu[tw] OR new hebrides[tw] OR venezuela[tw] OR vietnam[tw] OR viet nam[tw] OR middle east[tw] OR west bank[tw] OR gaza[tw] OR palestine[tw] OR yemen[tw] OR yugoslavia[tw] OR zambia[tw] OR zimbabwe[tw] OR northern rhodesia[tw] OR global south[tw] OR africa south of the sahara[tw] OR sub saharan africa[tw] OR subsaharan africa[tw] OR central africa[tw] OR north africa[tw] OR northern africa[tw] OR magreb[tw] OR maghrib[tw] OR sahara[tw] OR southern africa[tw] OR east africa[tw] OR eastern africa[tw] OR west africa[tw] OR western africa[tw] OR west indies[tw] OR indian ocean islands[tw] OR caribbean[tw] OR central america[tw] OR latin america[tw] OR south america[tw] OR central asia[tw] OR north asia[tw] OR northern asia[tw] OR southeastern asia[tw] OR south eastern asia[tw] OR southeast asia[tw] OR south east asia[tw] OR western asia[tw] OR east europe[tw] OR eastern europe[tw] OR developing country[tw] OR developing countries[tw] OR developing nation[tw] OR developing nations[tw] OR developing population[tw] OR developing populations[tw] OR developing world[tw] OR less developed country[tw] OR less developed countries[tw] OR less developed nation[tw] OR less developed nations[tw] OR less developed world[tw] OR lesser developed countries[tw] OR lesser developed nations[tw] OR under developed country[tw] OR under developed countries[tw] OR under developed nations[tw] OR under developed world[tw] OR underdeveloped country[tw] OR underdeveloped countries[tw] OR underdeveloped nation[tw] OR underdeveloped nations[tw] OR underdeveloped population[tw] OR underdeveloped populations[tw] OR underdeveloped world[tw] OR middle income country[tw] OR middle income countries[tw] OR middle income nation[tw] OR middle income nations[tw] OR middle income population[tw] OR middle income populations[tw] OR low income country[tw] OR low income countries[tw] OR low income nation[tw] OR low income nations[tw] OR low income population[tw] OR low income populations[tw] OR lower income country[tw] OR lower income countries[tw] OR lower income nations[tw] OR lower income population[tw] OR lower income populations[tw] OR underserved countries[tw] OR underserved nations[tw] OR underserved population[tw] OR underserved populations[tw] OR under served population[tw] OR under served populations[tw] OR deprived countries[tw] OR deprived population[tw] OR deprived populations[tw] OR poor country[tw] OR poor countries[tw] OR poor nation[tw] OR poor nations[tw] OR poor population[tw] OR poor populations[tw] OR poor world[tw] OR poorer countries[tw] OR poorer nations[tw] OR poorer population[tw] OR poorer populations[tw] OR developing economy[tw] OR developing economies[tw] OR less developed economy[tw] OR less developed economies[tw] OR underdeveloped economies[tw] OR middle income economy[tw] OR middle income economies[tw] OR low income economy[tw] OR low income economies[tw] OR lower income economies[tw] OR low gdp[tw] OR low gnp[tw] OR low gross domestic[tw] OR low gross national[tw] OR lower gdp[tw] OR lower gross domestic[tw] OR lmic[tw] OR lmics[tw] OR third world[tw] OR lami country[tw] OR lami countries[tw] OR transitional country[tw] OR transitional countries[tw] OR emerging economies[tw] OR emerging nation[tw] OR emerging nations[tw] OR afghanistan[mh] OR albania[mh] OR algeria[mh] OR american samoa[mh] OR angola[mh] OR antigua and barbuda[mh] OR argentina[mh] OR armenia[mh] OR aruba[mh] OR azerbaijan[mh] OR bahrain[mh] OR bangladesh[mh] OR barbados[mh] OR republic of belarus[mh] OR belize[mh] OR benin[mh] OR bhutan[mh] OR bolivia[mh] OR bosnia and herzegovina[mh] OR botswana[mh] OR brazil[mh] OR bulgaria[mh] OR burkina faso[mh] OR burundi[mh] OR cabo verde[mh] OR cambodia[mh] OR cameroon[mh] OR central african republic[mh] OR chad[mh] OR chile[mh] OR china[mh] OR colombia[mh] OR comoros[mh] OR democratic republic of the congo[mh] OR congo[mh] OR costa rica[mh] OR cote d'ivoire[mh] OR croatia[mh] OR cuba[mh] OR cyprus[mh] OR czech republic[mh] OR djibouti[mh] OR dominica[mh] OR dominican republic[mh] OR ecuador[mh] OR egypt[mh] OR el salvador[mh] OR equatorial guinea[mh] OR eritrea[mh] OR estonia[mh] OR eswatini[mh] OR ethiopia[mh] OR fiji[mh] OR gabon[mh] OR gambia[mh] OR "georgia republic"[mh] OR ghana[mh] OR gibraltar[mh] OR greece[mh] OR grenada[mh] OR guam[mh] OR guatemala[mh] OR guinea[mh] OR guinea-bissau[mh] OR guyana[mh] OR haiti[mh] OR honduras[mh] OR hungary[mh] OR india[mh] OR indonesia[mh] OR iran[mh] OR iraq[mh] OR jamaica[mh] OR jordan[mh] OR kazakhstan[mh] OR kenya[mh] OR democratic people's republic of korea[mh] OR kosovo[mh] OR kyrgyzstan[mh] OR laos[mh] OR latvia[mh] OR lebanon[mh] OR lesotho[mh] OR liberia[mh] OR libya[mh] OR lithuania[mh] OR macau[mh] OR republic of north macedonia[mh] OR madagascar[mh] OR malawi[mh] OR malaysia[mh] OR indian ocean islands[mh] OR mali[mh] OR malta[mh] OR micronesia[mh] OR palau[mh] OR mauritania[mh] OR mauritius[mh] OR mexico[mh] OR moldova[mh] OR mongolia[mh] OR montenegro[mh] OR morocco[mh] OR mozambique[mh] OR myanmar[mh] OR namibia[mh] OR nepal[mh] OR netherlands antilles[mh] OR nicaragua[mh] OR niger[mh] OR nigeria[mh] OR oman[mh] OR pakistan[mh] OR panama[mh] OR papua new guinea[mh] OR paraguay[mh] OR peru[mh] OR philippines[mh] OR poland[mh] OR portugal[mh] OR puerto rico[mh] OR romania[mh] OR russia[mh] OR rwanda[mh] OR samoa[mh] OR sao tome and principe[mh] OR saudi arabia[mh] OR senegal[mh] OR serbia[mh] OR seychelles[mh] OR sierra leone[mh] OR slovakia[mh] OR slovenia[mh] OR melanesia[mh] OR somalia[mh] OR south africa[mh] OR south sudan[mh] OR sri lanka[mh] OR saint kitts and nevis[mh] OR saint lucia[mh] OR saint vincent and the grenadines[mh] OR sudan[mh] OR suriname[mh] OR syria[mh] OR tajikistan[mh] OR tanzania[mh] OR thailand[mh] OR timor-leste[mh] OR togo[mh] OR tonga[mh] OR trinidad and tobago[mh] OR tunisia[mh] OR turkey[mh] OR turkmenistan[mh] OR uganda[mh] OR ukraine[mh] OR uruguay[mh] OR uzbekistan[mh] OR vanuatu[mh] OR venezuela[mh] OR vietnam[mh] OR middle east[mh] OR yemen[mh] OR yugoslavia[mh] OR zambia[mh] OR zimbabwe[mh] OR africa south of the sahara[mh] OR africa, central[mh] OR africa, northern[mh] OR africa, southern[mh] OR africa, eastern[mh] OR africa, western[mh] OR west indies[mh] OR indian ocean islands[mh] OR caribbean region[mh] OR central america[mh] OR latin america[mh] OR south america[mh] OR asia, central[mh] OR asia, northern[mh] OR asia, southeastern[mh] OR asia, western[mh] OR europe, eastern[mh] OR developing countries[mh])

**References**

1 Bautista MAC, Nurjono M, Lim YW, *et al.* Instruments Measuring Integrated Care: A Systematic Review of Measurement Properties. *Milbank Quarterly* 2016;**94**:862–917. doi:10.1111/1468-0009.12233

2 LMIC Filters. Cochrane Effective Practice and Organisation of Care. 2020.https://epoc.cochrane.org/lmic-filters (accessed 27 Apr 2022).
